# Stomach 3D Reconstruction Using Virtual Chromoendoscopic Images

**DOI:** 10.1109/JTEHM.2021.3062226

**Published:** 2021-02-24

**Authors:** Aji Resindra Widya, Yusuke Monno, Masatoshi Okutomi, Sho Suzuki, Takuji Gotoda, Kenji Miki

**Affiliations:** 1 Department of Systems and Control EngineeringSchool of EngineeringTokyo Institute of Technology 13290 Tokyo152-8550Japan; 2 Division of Gastroenterology and HepatologyDepartment of MedicineNihon University School of MedicineTokyo101-8309Japan; 3 Department of Internal MedicineTsujinaka Hospital KashiwanohaKashiwa277-0871Japan

**Keywords:** Endoscopy, stomach, 3D reconstruction, structure-from-motion, generative adversarial network

## Abstract

Gastric endoscopy is a golden standard in the clinical process that enables medical practitioners to diagnose various lesions inside a patient’s stomach. If a lesion is found, a success in identifying the location of the found lesion relative to the global view of the stomach will lead to better decision making for the next clinical treatment. Our previous research showed that the lesion localization could be achieved by reconstructing the whole stomach shape from chromoendoscopic indigo carmine (IC) dye-sprayed images using a structure-from-motion (SfM) pipeline. However, spraying the IC dye to the whole stomach requires additional time, which is not desirable for both patients and practitioners. Our objective is to propose an alternative way to achieve whole stomach 3D reconstruction without the need of the IC dye. We generate virtual IC-sprayed (VIC) images based on image-to-image style translation trained on unpaired real no-IC and IC-sprayed images, where we have investigated the effect of input and output color channel selection for generating the VIC images. We validate our reconstruction results by comparing them with the results using real IC-sprayed images and confirm that the obtained stomach 3D structures are comparable to each other. We also propose a local reconstruction technique to obtain a more detailed surface and texture around an interesting region. The proposed method achieves the whole stomach reconstruction without the need of real IC dye using SfM. We have found that translating no-IC green-channel images to IC-sprayed red-channel images gives the best SfM reconstruction result. Clinical impact We offer a method of the frame localization and local 3D reconstruction of a found gastric lesion using standard endoscopy images, leading to better clinical decision.

## I. Introduction

Gastric endoscopy is a well-applied clinical process that enables medical practitioners to find a gastric lesion, such as an ulcer and cancer, inside the patient’s stomach. The accurate localization of a found malignant lesion is very important to decide the next clinical procedure. For example, if laparoscopic gastroectomy for early cancer needs to be performed, the target cancer location relative to the global view of the stomach should be known to decide the operative procedure. The successful localization of a found malignant lesion leads to better and more effective decision making by the doctors. However, accurately recognizing the lesion’s 3D location only from 2D endoscopic images is difficult for gastric surgeons, especially when the images are captured by another endoscopist.

To address the difficulty of the lesion localization in gastric endoscopy, previous studies propose some 2D or 3D approaches. The examples of the 2D approach are X-ray barium radiography [1] which is able to reveal various characteristics of the stomach tract and view expansion [2], [3] which provides panoramic views for broader sight. However, they only provide 2D information which is not sufficient for the localization of a lesion with morphological change. As a more sophisticated approach, 3D computed tomography [4] performs the 3D reconstruction of a whole stomach which provides better morphological information. However, the lack of color and texture information in the 3D computed tomography makes the lesion inspection and localization difficult, especially for flat lesions without morphological change.

Various vision-based organ 3D reconstruction methods based on Shape-from-shading (SfS) [5]–[7], Visual Simultaneous Localization and Mapping (SLAM) [8]–[10], Structure-from-Motion (SfM) [11]–[13], and monocular depth estimation [14], [15], which are able to recover both 3D structure and color-texture information, have also been proposed. However, existing works mainly focus on the partial surface reconstruction of a target organ, aiming for lesion inspection and surgery applications (see [16]–[18] for surveys). The research of whole organ 3D reconstruction from endoscopy images is still limited for the lesion localization in endoscopy.

**FIGURE 1.**
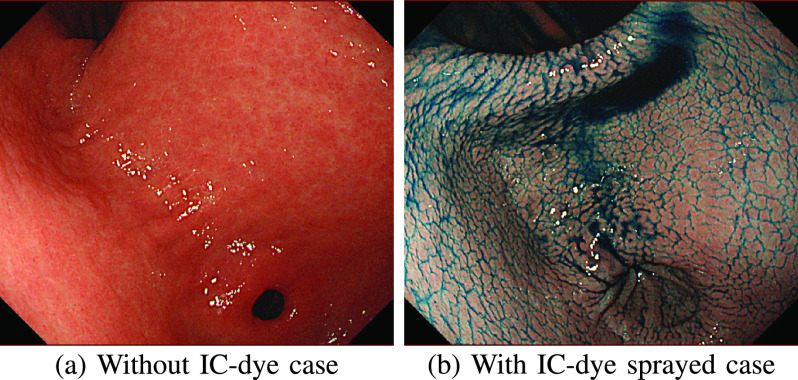
A visual comparison between the stomach surface images without IC-dye and with IC-dye sprayed. The image (a) shows a very smooth and texture-less surface which makes feature extraction and matching processes difficult, while the image (b) shows more visible textures which can be extracted for SfM.

In our previous study, we tackled the drawbacks of the previous studies for lesion localization by reconstructing the color-textured 3D model of a whole stomach from an endoscope video based on an SfM pipeline [19], [20]. Although the stomach 3D reconstruction by SfM is very challenging because of texture-less stomach surfaces as shown in Figure 1(a), we found that the whole stomach shape can be reconstructed by using red-channel images of chromoendoscopy with indigo carmine (IC) blue dye, where the IC dye acts as an enhancement substance to bring up more textures to the stomach surface as shown in Figure 1(b). However, though the IC dye is commonly used in gastric endoscopy [21], [22], spraying it on the whole stomach surface requires additional procedure time, labor, and cost, which is not desirable for both patients and medical practitioners. Furthermore, the IC dye may hinder the visibility of the reconstructed stomach surface because of its dark color tone.

In this paper, we propose a novel SfM-based approach for whole stomach 3D reconstruction that does not require to capture chromoendoscopic image sequences. Instead of spraying the IC dye during endoscopy, we generate virtual IC-dye-sprayed (VIC) images from no-IC images based on image-to-image style translation with a cycle-consistent generative adversarial network (CycleGAN) [23]. The SfM pipeline is then applied using the generated VIC images.

With the rise of deep learning, image-to-image style translation, in which the goal is to learn the mapping between one style of images to another, is attracting attention from researchers. The style translation has been proven to be useful for endoscopy applications such as in colonoscopy depth estimation [24]–[26]. It is also reported that generating VIC images improves the lesion detection and classification performance in colonoscopy [27]. Inspired by the study in [27], we propose VIC image generation for stomach 3D reconstruction, which is, to the best of our knowledge, firstly reported in this paper.

In our experiments, we trained several CycleGANs for the style translation using different input and output color channel pairs and found that CycleGAN translating the no-IC green-channel images to the IC-sprayed red-channel images gives the best VIC images for SfM. Using the generated VIC images, we were able to reconstruct the whole stomach 3D model without the need of real IC-sprayed images. We also confirmed that, using the reconstructed 3D model and the estimated camera poses, image frame localization can be performed to identify the 3D location of an interesting region.

This paper is an extended version of our previous work published in [28]. In this paper, we provide more detailed explanation on the image-to-image translation process. We also explain a new frame localization and local 3D mesh refinement pipeline. Furthermore, we demonstrate additional experimental results for both subjective and objective evaluation showing the advantages of our proposed approach. Finally, we demonstrate additional validation results of our 3D models reconstructed using generated VIC images by comparing them with the 3D models reconstructed using real IC-sprayed images.

The rest of this paper is organized as follows. Section II details our endoscope video dataset and proposed pipeline. Section III shows our experimental results and provides the discussion on them. Finally, Section IV concludes the paper.

## II. Materials and Methods

**FIGURE 2.**
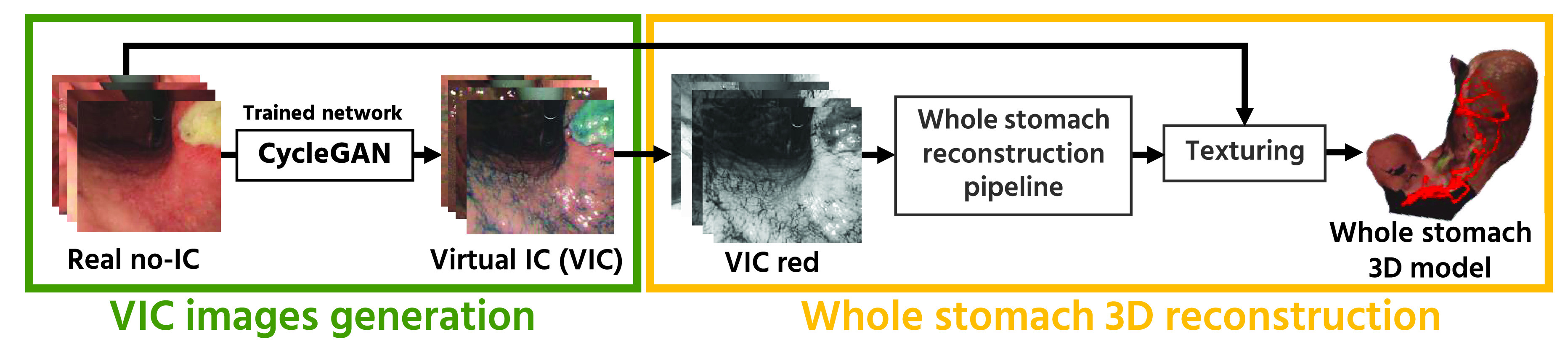
The overview of our proposed pipeline. Our proposed pipeline consists of the VIC images generation using a separately trained CycleGAN and the whole stomach 3D reconstruction using generated VIC red-channel images. In this work, we trained three CycleGANs illustrated in Figure 3 and investigated which domain pair produces better 3D reconstruction results.

Figure 2 illustrates the overview of our proposed whole stomach 3D reconstruction pipeline. In this section, we explain the detail of the proposed pipeline including our endoscope video dataset (Section II-A), CycleGAN model (Section II-B), VIC generation (Section II-C), 3D reconstruction pipeline (Section II-D), and local 3D mesh refinement pipeline (Section II-E).

### A. Endoscope Video Dataset

In this work, we used exactly the same endoscope video dataset from our previous work [20]. Seven videos captured from seven subjects undergoing general gastroendoscopy procedure are included in the dataset. To account for the patient body and stomach peristalsis movement, a sedative drug and antispasmodic were used. Each video contains two different image type sequences: no-IC and IC-sprayed sequences. We extracted the image frames from each video and divided them into no-IC images and IC-sprayed images to obtain training image data for VIC images generation. We also extracted test no-IC image sequences for the 3D reconstruction. Each of the test no-IC image sequences is a one-pass sequence which means that one-way trajectory of the endoscope covering top-to-bottom or bottom-to-top of the stomach is included in the sequence. The number of input no-IC image frames for the 3D reconstruction can be found in Table 1 of the experimental result section.

**TABLE 1:** The Objective Evaluation of SfM Results. The no-IC Green Case is the Baseline Compared to VIC Red Cases

		Subject A	Subject B	Subject C	Subject D	Subject E	Subject F	Subject G
	Input images	2302	439	1715	829	1726	1901	1297
No-IC green images (baseline)	Reconstructed images	2165 (94.05%)	217 (49.43%)	54 (3.15%)	288 (34.74%)	497 (28.79%)	47 (2.47%)	1248 (96.22%)
	3D points	2,043,78	13,568	2,410	14,678	27,617	2,759	105,691
	Avg. observations	684.18	524.67	260.35	353.31	368.70	339.77	650.97
VIC red images from cGAN_rgb2rgb_	Reconstructed images	272 (11.82%)	166 (37.81%)	873 (50.90%)	98 (11.82%)	263 (11.24%)	615 (32.35%)	328 (25.29%)
	3D points	11,323	10,326	62,556	4,020	11,973	38,224	20,396
	Avg. observations	228.01	475.21	450.55	295.74	224.36	372.26	406.77
VIC red images from cGAN_r2r_	Reconstructed images	270 (11.73%)	389 (88.61%)	535 (31.20%)	376 (45.36%)	1410 (81.69%)	1099 (57.81%)	1297 (100%)
	3D points	25,756	41,395	71,437	37,610	160,345	110,002	157,940
	Avg. observations	507.98	816.29	797.40	637.51	662.17	593.45	814.64
VIC red images from cGAN_g2r_	Reconstructed images	2201 (95.61%)	438 (99.77%)	1662 (96.91%)	823 (99.28%)	1668 (96.64%)	1838 (96.69%)	1297 (100%)
	3D points	412,089	44,866	207,795	100,115	238,285	231,744	213,249
	Avg. observations	2127.81	1007.01	1111.10	1099.95	1188.62	881.70	1504.79

**Ethics.** This study was conducted in accordance with the Declaration of Helsinki and approved by the Institutional Review Board at Nihon University Hospital on March 8, 2018 (Identification No.: 180302) and Tokyo Institute of Technology on March 30, 2018 (Identification No.: 2017125). Informed consent was obtained from all enrolled subjects. This study was registered with the University Hospital Medical Information Network (UMIN) Clinical Trials Registry on March 17, 2018. (Identification No.: UMIN000031776).

### B. Cycle-Consistent Image-to-Image Translation (CycleGAN)

Since the capture time of the no-IC and IC-sprayed sequences are different, it is impossible to obtain the exact pair between those types of images. Because of that, we apply CycleGAN [23] as our image-to-image translator because CycleGAN works with unsupervised and unpaired training data. Let $A$ and $B$ be two different image domains. CycleGAN consists of two sets of generator and discriminator pair, $(G_{A}, D_{A})$ and $(G_{B}, D_{B})$. The generator’s task is to generate a virtual image by translating an input image from one domain to another and fool its opposite domain’s discriminator. On the other hand, the discriminator’s task is to distinguish the generated and the real images. For example, the generator $G_{A}$’s task is to translate an image from domain $A$ to domain $B$ and fool the discriminator $D_{B}$.

The total loss of CycleGAN consists of two least-square GAN losses [29], cycle consistent loss, and identity loss. The total loss can be expressed as:

\begin{align*} \mathcal {L}(G_{A},G_{B},D_{A},D_{B})=&\mathcal {L}_{GAN}(G_{A},D_{B},A,B) \\&+ \mathcal {L}_{GAN}(G_{B},D_{A},A,B) \\&+ \lambda _{cyc}\mathcal {L}_{cyc}(G_{A},G_{B}) \\&+ \lambda _{idt}\mathcal {L}_{idt}(G_{A},G_{B})\tag{1}\end{align*}

The GAN loss describes the competition between a pair of a generator and a discriminator. The first GAN loss, which expresses the generator-discriminator competition in $A \rightarrow B$ direction, can be formulated as follows:

\begin{align*} \mathcal {L}_{GAN}(G_{A},D_{B},A,B) = &\mathbb {E}_{b\sim p_{data}(b)}[(D_{B}(b)-1)^{2}] \\& \,\, + \mathbb {E}_{a\sim p_{data}(a)}[(D_{B}(G_{A}(a)))^{2}] \tag{2}\end{align*}

In this translation direction, the generator $G_{A}$ tries to generate image $b' = G_{A}(a)$ from a randomly sampled image $a \sim p_{data}(a)$. The discriminator $D_{B}$ then tries to distinguish between the generated image $b'$ and a randomly sampled real image $b \sim p_{data}(b)$. Based on the loss of (2), the discriminator $D_{B}$ is trained to give a high score for the real image $b$ and a low score for the generated image $b'$, while the generator $G_{A}$ is trained to fool the discriminator $D_{B}$. The same principle also applies for the opposite direction, i.e., $B \rightarrow A$ direction. Therefore, CycleGAN has two GAN losses.

The consistency loss makes sure that CycleGAN is able to generate an image that is as close as possible to its input image when translating it circularly, i.e., $a \approx G_{B}(G_{A}(a))$. Following the previous notation, the cycle consistency loss can be formulated as follows:

\begin{align*} \mathcal {L}_{cyc}(G_{A},G_{B}) = &\mathbb {E}_{a\sim p_{data}(a)}\big [\big \|G_{B}(G_{A}(a)) - a\big \|_{1}\big] \\& \quad + \mathbb {E}_{b\sim p_{data}(b)}\big [\big \|G_{A}(G_{B}(b)) - b\big \|_{1}\big]\tag{3}\end{align*}

The consistency loss enables CycleGAN to be trained on the unpaired set of images for image-to-image style translation.

Finally, the identity loss is added to prevent the mapping when a real sample from the target domain is fed as an input to the generator. The identity loss is expressed as follows:

\begin{align*} \mathcal {L}_{idt}(G_{A},G_{B}) = &\mathbb {E}_{b\sim p_{data}(b)}\big [\big \|G_{A}(b) - b\big \|_{1}\big] \\& \quad + \mathbb {E}_{a\sim p_{data}(a)}\big [\big \|G_{B}(a) - a\big \|_{1}\big]\tag{4}\end{align*}

In the training time, the degrees of importance for the cycle consistency and the identity losses are determined by $\lambda _{cyc}$ and $\lambda _{idt}$.

### C. VIC Images Generation Using CycleGAN

**FIGURE 3.**
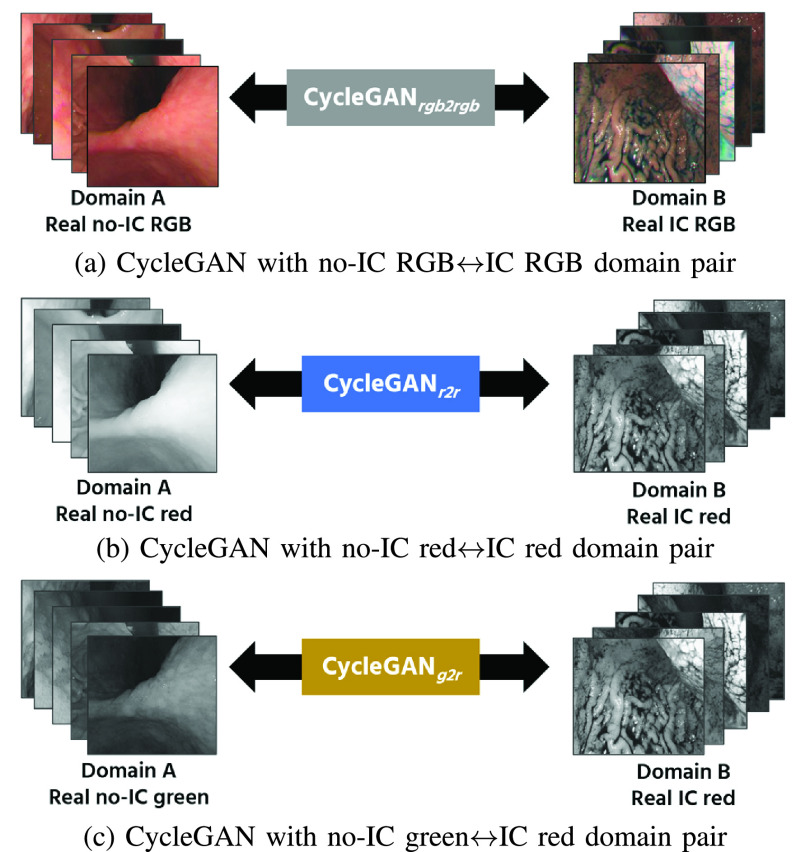
The overview of our CycleGAN training. We train three CycleGANs with different domain pairs, i.e., (a) No-IC RGB$\leftrightarrow $IC-sprayed RGB, (b) No-IC red$\leftrightarrow $IC-sprayed red, and (c) No-IC green$\leftrightarrow $IC-sprayed red. We then investigate which domain pair gives the best 3D reconstruction result for SfM. We describe detailed explanation about the domain pair selection in Section II-C.

Figure 3 shows our CycleGAN training overview. We train CycleGAN to learn the mapping between no-IC images (domain A) and IC-sprayed images (domain B) for VIC images generation. For the CycleGAN training, we use both real no-IC and real IC-sprayed images extracted from the endoscope video dataset.

In our previous research, we observed that there is a color channel misalignment, which means that R, G, and B channel images of one RGB image are not perfectly aligned. This is caused by the imperfection of the color image generation by the endoscope system, which combines sequentially captured R, G, and B images to form one RGB image. The color channel misalignment causes some texture patterns to appear duplicated and disturbs the SfM pipeline (See Figure 1 in [20]). Because of that, we used single-channel images for SfM and investigated which color channel gives the best 3D reconstruction result. It was found that the whole stomach can be reconstructed using IC-sprayed red-channel images because the red channel of IC-sprayed images has the best contrast and the most visible textures among the other channels. It was also found that, for the case of no-IC images, the green channel gives the best 3D reconstruction result, though only partial stomach could be reconstructed. The blue channel was not preferable for the 3D reconstruction due to low contrasts.

Based on the above findings, we use the VIC red-channel images as SfM inputs for the 3D reconstruction. To effectively generate the VIC red images, we investigate the results of three CycleGANs with different channel domain pairs. Specifically, we set the domain pair, $A$ and $B$, for each CycleGAN to the following pairs: (i) No-IC RGB and IC-sprayed RGB image domain pair (Figure 3(a)). This pair is considered because the RGB-to-RGB translation is the common practice for the image-to-image translation. Since we use the VIC red images for SfM inputs, we extract the red-channel images from the RGB-to-RGB translation results in the subsequent processes. (ii) No-IC red and IC-sprayed red image domain pair (Figure 3(b)). This pair uses the red channel for both input and output domains, which can be cosidered as one of the most straightforward ways to generate the VIC red images. (iii) No-IC green and IC-sprayed red image domain pair (Figure 3(c)). This pair uses the green channel for the input domain because no-IC green images achieve the most complete SfM result for the no-IC case. In this domain pair setting, we pair the color channels that achieve the best 3D reconstruction for no-IC and IC-sprayed image sequences, respectively. For the rest of this paper, we will refer to each CycleGAN as cGAN_rgb2rgb_, cGAN_r2r_, and cGAN_g2r_ respectively. After the training process, the VIC red images are generated from no-IC images using each of the trained CycleGANs.

### D. 3D Reconstruction Using the Generated VIC Red Images

Using the generated VIC red images, we follow the 3D reconstruction pipeline presented in our previous research [20]. It consists of point cloud reconstruction, outlier removal, and mesh and texture generation. The point cloud reconstruction follows the general flow of SfM [32]. It starts with detecting and extracting the Scale Invariant Feature Transform (SIFT) features [33] from all of the input images. Then, exhaustive feature matching across the input frames is performed using the extracted SIFT features. Those steps are then followed by features triangulation, poses estimation, and bundle adjustment [34] in parallel. It is then followed by random sample consensus (RANSAC)-based plane-fitting outlier removal to remove apparent outlier 3D points. After removing the outlier points, Poisson surface reconstruction [35] is applied to obtain a triangle mesh model. Finally, the triangle mesh is textured using the original no-IC RGB images by the method of [30], [36]. As the final result, our entire pipeline produces a textured triangle mesh of the stomach.

### E. Local Mesh Refinement for a Localized Frame

**FIGURE 4.**
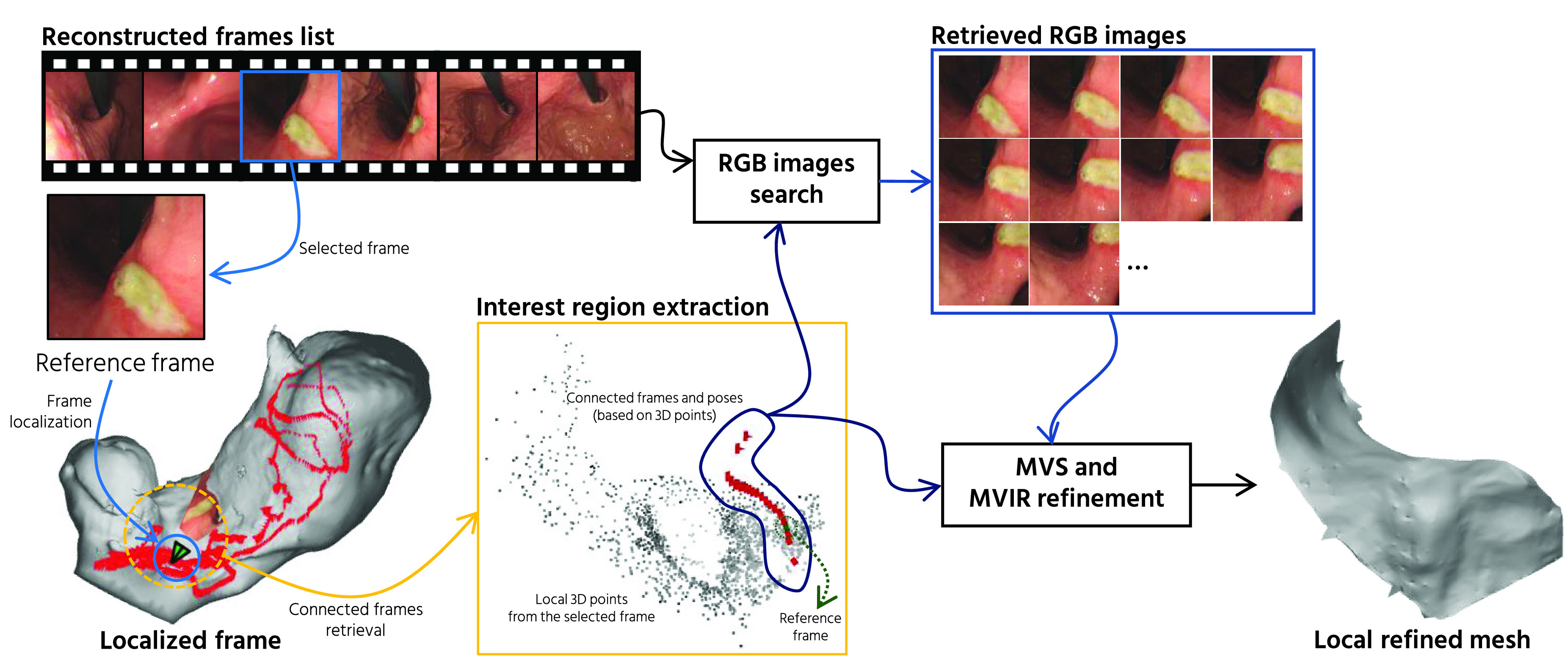
The flow of our proposed frame localization and local mesh refinement fot the localized region. Firstly, the frame of interest is selected from the list of reconstructed frames. After the selected reference frame is localized by the camera pose retrieval process, the selected frame texture is projected to the reconstructed mesh. After that, local mesh refinement is performed by retrieving $N$ number of the RGB images with the camera poses that see the same 3D points originated from the reference frame. Using the retrived RGB images and camera poses, MVS [30] and MVIR [31] are applied for the mesh refinement.

After we reconstruct the whole stomach 3D model, we perform the frame localization of an interesting frame and local mesh refinement for the localized region. Figure 4 illustrates our proposed frame localization and local mesh refinement pipeline. Our frame localization accepts a selected frame from the reconstructed frames list as an input. Then, the frame localization is performed by retrieving the camera pose of the selected frame and projecting the no-IC RGB image texture to the corresponding reconstructed mesh.

After the selected frame is localized, it is desirable to acquire a more focused view of the stomach surface. To provide a more detailed local reconstruction result, we propose a new local mesh refinement pipeline that makes use of the already reconstructed whole stomach model. To perform refined local reconstruction, we first obtain the 3D points from the point cloud that originate from the selected reference frame. To obtain a higher quality mesh, we then retrieve $N$ number of frames connected from the reference frame using the track information of the obtained local 3D points. The corresponding RGB images of the connected cameras are then retrieved from the set of the reconstructed frames. Instead of applying Poisson surface reconstruction [35], we use the locally connected camera poses and the corresponding RGB images as the inputs for Multi-View Stereo (MVS) [30], [37]. Then we further refine the MVS result with Multiv-View Inverse Rendering (MVIR) [31]. The output mesh of MVIR is used for the texturing using no-IC RGB images.

## III. Results and Discussion

### A. Implementation Details

We individually trained each CycleGAN using a single NVIDIA GeForce GTX 1080Ti GPU. Following the original CycleGAN [23], we used 9 blocks of ResNet [38] for our generator network and three layers of PatchGAN [39] for our discriminator network. We set the weights for cycle consistency and identity losses in (1) to $\lambda _{cyc}=10$ and $\lambda _{idt}=5$, respectively. The network was trained for 100 epochs for each domain pair setting, i.e., cGAN_rgb2rgb_, cGAN_r2r_, and cGAN_g2r_ using the training data of 7978 no-IC images and 7453 IC-sprayed images. Due to the GPU memory limitation, we resized the original $1155\times 1003$ images to $600\times 524$ pixels and trained the CycleGANs with randomly cropped image patches of $510\times 510$ pixels. The training for each domain pair took around 100 hours to complete. For the 3D reconstruction pipeline, we used the same setup and implementation as our previous research [20]. For the local mesh refinement, we extracted $N=22$ connected images from the global reconstruction as the inputs for the refinement.

### B. VIC Image Generation Results

**FIGURE 5.**
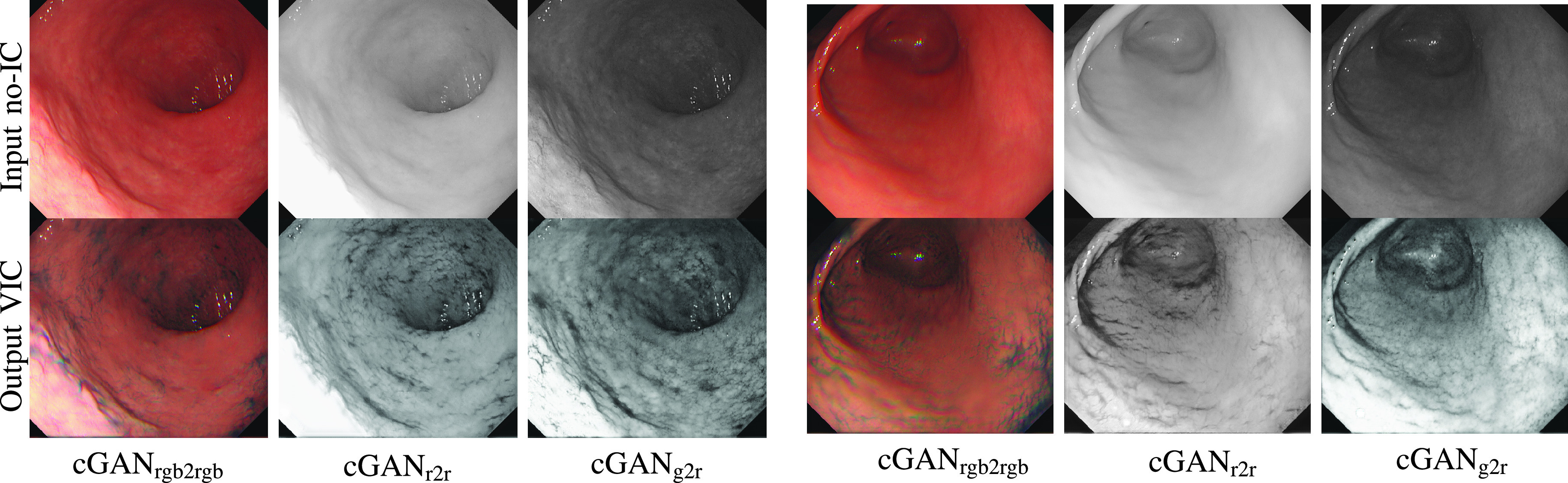
Example results of the generated VIC images. The top row shows the input no-IC images and the bottom row shows the corresponding generated VIC images. From left to right in each group, we show the translation results of no-IC RGB $\rightarrow $ VIC RGB with cGAN_rgb2rgb_, no-IC red $\rightarrow $ VIC red with cGAN_r2r_, and no-IC green $\rightarrow $ VIC red with cGAN_g2r_, respectively. We can see that each of CycleGAN successfully generates the VIC image which has more visible textures compared to the texture-less surface of the no-IC image.

We first show the example results of generated VIC images using cGAN_rgb2rgb_, cGAN_r2r_, and cGAN_g2r_. Figure 5 shows the comparison between the input no-IC images and the generated VIC images using each of the trained CycleGAN. As we can see from the results, all CycleGANs were able to generate VIC image by transferring the pattern and contrast styles of the IC-sprayed image to the input no-IC image. However, if we see the no-IC red-channel images (top row of the second and fifth columns), we can observe that the stomach surface is fairly texture-less. Even for convolutional neural networks, it is hard to extract features from this kind of texture-less images. On the other hand, the no-IC green images (top row of third and sixth columns) show more textures, enabling slightly better style transfer. We also show the examples of the generated VIC RGB images using cGAN_rgb2rgb_ on the first and fourth columns. From the RGB-to-RGB translation examples, we can observe that the color channel misalignment problem is carried out by the network, which makes the translation is not ideal. In the following subsection, we discuss the effect of the input channel selection on feature matching for SfM.

### C. Feature Matching Results

After generating the VIC images of all sequences from the seven subjects, we calculated the average number of extracted SIFT features per image. For the VIC image from cGAN_rgb2rgb_, we extracted its red-channel for the feature extraction. The VIC red images from cGAN_r2r_, cGAN_g2r_, and cGAN_rgb2rgb_ have the average number of 3401.70, 3346.94, and 4218.19 extracted features, respectively. As the baselines, we also calculated the average numbers of extracted features of no-IC red and no-IC green images, which are 614.06 and 889.66 features, respectively. It is clear that the VIC images have more extracted features compared to the no-IC images by more than four times.

However, solely increasing the number of features is not sufficient. Since SfM relies on the consistency of extracted features across multiple images, we also tested the feature matching performance of the generated VIC images. For this purpose, we extracted 11 consecutive images from a sequence. We then used the first image as an anchor, $t$, and performed feature matching to all of its consecutive images, $t+1, t+2, \ldots, t+10$.

**FIGURE 6.**
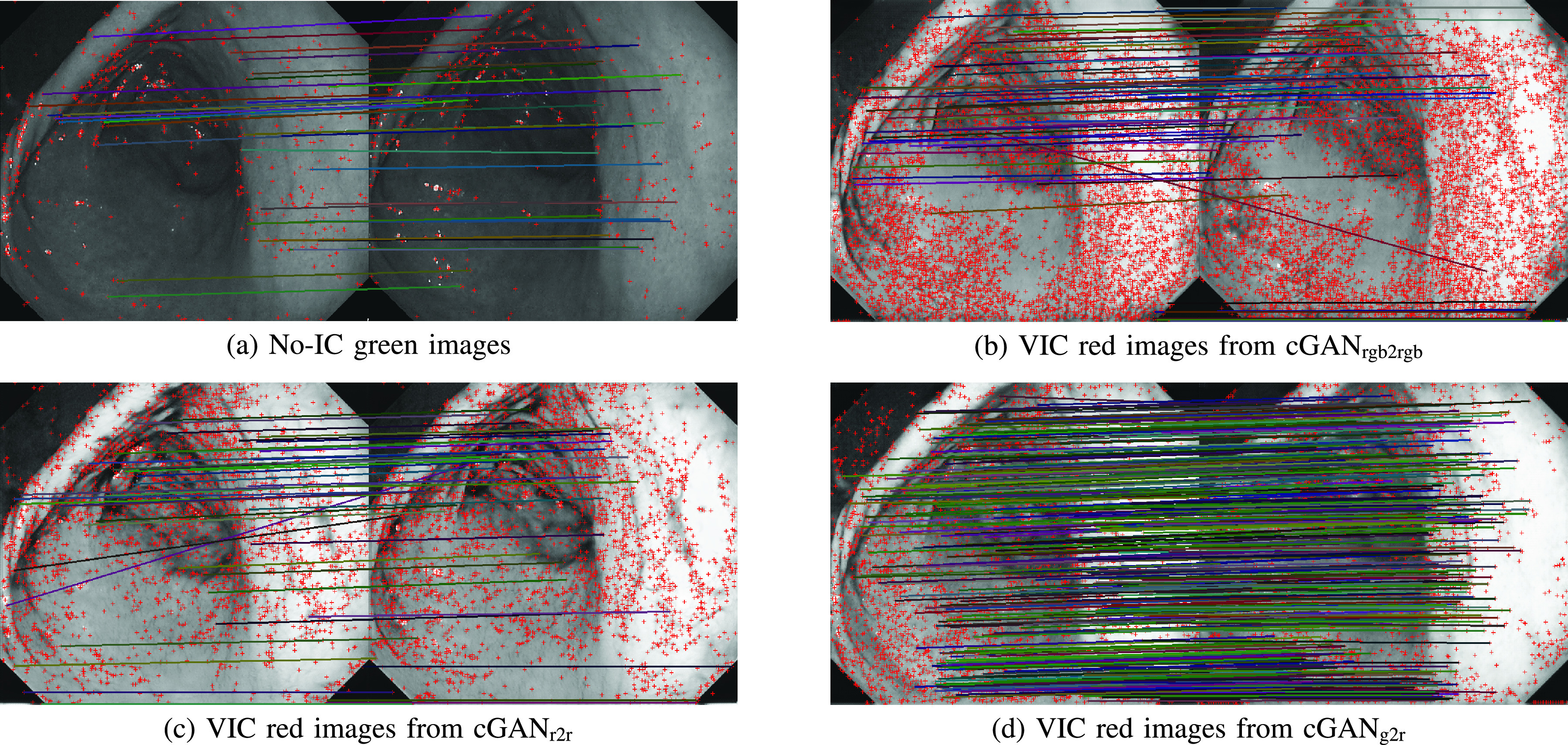
The example of inlier feature matching results for two frames ($t$ and $t+9$). The red marks represent the locations of extracted SIFT features. The color lines represent the matched features. It is clear that the number of feature matches in (b) and (c) is much fewer than that in (d), even though the number of extracted features in (b) and (c) significantly increases from (a). This result implies that the generated VIC red images from cGAN_g2r_ have better pattern consistency between the frames.

**FIGURE 7.**
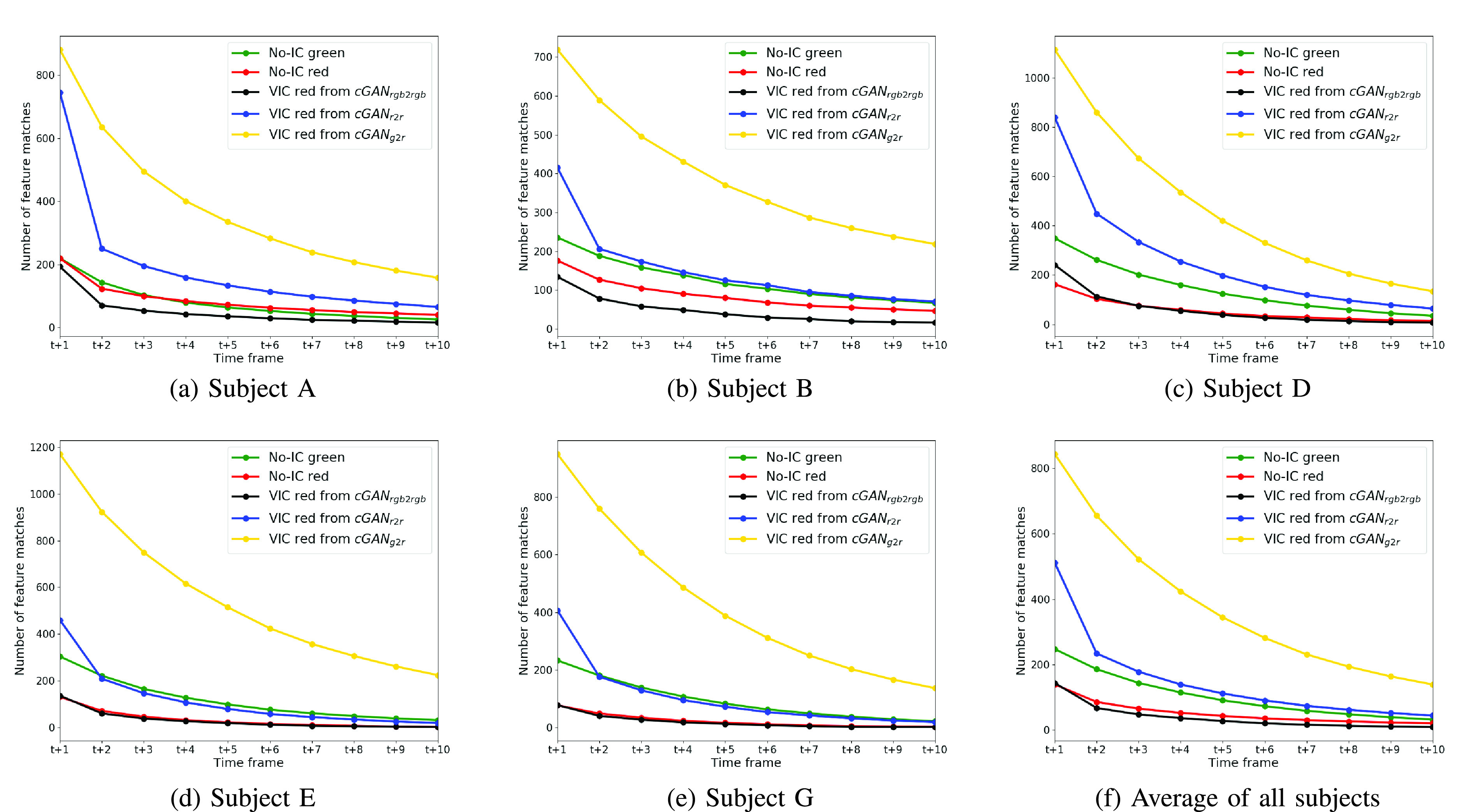
Comparison of the average number of feature matches between the anchor frame and its 10 consecutive frames. The x-axis represents the relative time stamp and the y-axis represents the average number of feature matches calculated for every 10 consecutive frames. It is clearly shown that the VIC images from cGAN_g2r_ has a higher number of feature matches across the frames.

Figure 6 shows the example feature matching results. Even though cGAN_rgb2rgb_ has the highest average number of extracted SIFT features, it can be seen that the feature matching performance is similar to the no-IC green image case. It is because that there is color channel misalignment in the RGB image. Figure 7 shows the average number of feature matches between the anchor frame and each of its consecutive frames taken from group-of-11-consecutive-images samples, which were extracted from the Subject A, B, D, E, and G. We also show the average number of feature matches for all seven subjects used in our experiment. It can be seen that the VIC red images from cGAN_g2r_ has a higher number of matches across frames compared to the other four image types. We can also see that even the VIC images from cGAN_r2r_ results has a high number of matches for *t vs t* + *1*, the number of matches drops significantly for the following frames. It implicitly means that the VIC red images from cGAN_g2r_ has better temporal pattern consistency between frames.

### D. 3D Reconstruction Results

**FIGURE 8.**
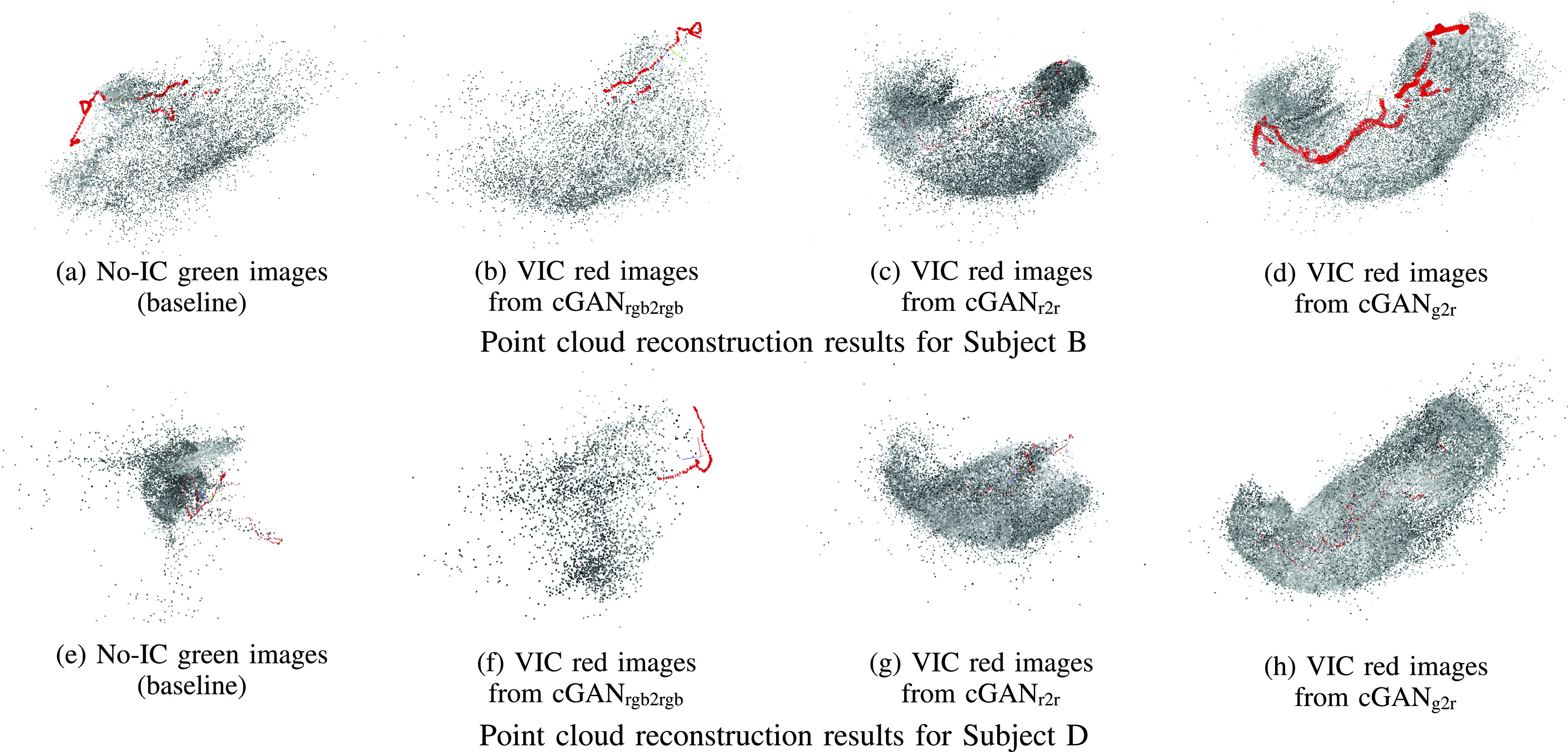
The SfM reconstruction results of Subject B (top) and Subject D (bottom) using no-IC green images (first column), VIC red images from cGAN_rgb2rgb_ (second column), VIC red images from cGAN_r2r_ (third column), and VIC red images from cGAN_g2r_ (fourth column). The gray dots represent the reconstructed 3D points and the red pyramids represent the estimated camera poses. Significant improvements from the baseline results of (a) and (e) are shown by the results of (d) and (h) using the VIC red images from cGAN_g2r_.

Since our proposed pipeline is based on SfM [32], all the input frames should be available prior to the start of the reconstruction. In other words, our reconstruction pipeline can only work in an offline manner. Figure 8 shows the SfM reconstruction results for Subject B and D using four different image types, i.e., no-IC green images, VIC red images from cGAN_rgb2rgb_, VIC red images from cGAN_r2r_, and VIC red images from cGAN_g2r_. Since all the mentioned types of images were extracted and generated from the same source RGB sequence, the comparison can be fairly performed. Using those types of images, 49.43%, 37.81%, 88.84%, and 99.77% images of Subject B and 34.74%, 11.82%, 81.69%, and 99.28% images of Subject D were reconstructed, respectively. In Figure 8(a) and (e), the stomach shape cannot be reconstructed using no-IC green images. In Figure 8(b) and (f), the results using VIC red images from cGAN_rgb2rgb_ also shows incomplete reconstruction results. Moreover, these results are worse than the baseline no-IC green case, which can be considered by the channel misalignment problem in the RGB images. In Figure 8(c) and (g), the results using VIC red images from cGAN_r2r_ only show partially reconstructed stomach shapes. In Figure 8(d) and (h), we can confirm that the results using VIC red images from cGAN_g2r_ achieve the best point cloud quality and completeness.

Table 1 shows the objective evaluation of SfM reconstruction results on all seven subjects. It shows that the generated VIC red images from cGAN_g2r_ achieve better results on all subjects compared to the baseline no-IC green images. Using the VIC red from cGAN_g2r_ for SfM significantly improves the number of reconstructed images, especially for Subject B to F. All reconstruction results using the VIC red from cGAN_g2r_ achieve more than 95% of reconstructed images. Since the number of feature matches that can be maintained across multiple frames are higher in VIC red from cGAN_g2r_, it leads to the increase of features that could be triangulated, as shown by “Avg. observation” in the table.

**FIGURE 9.**
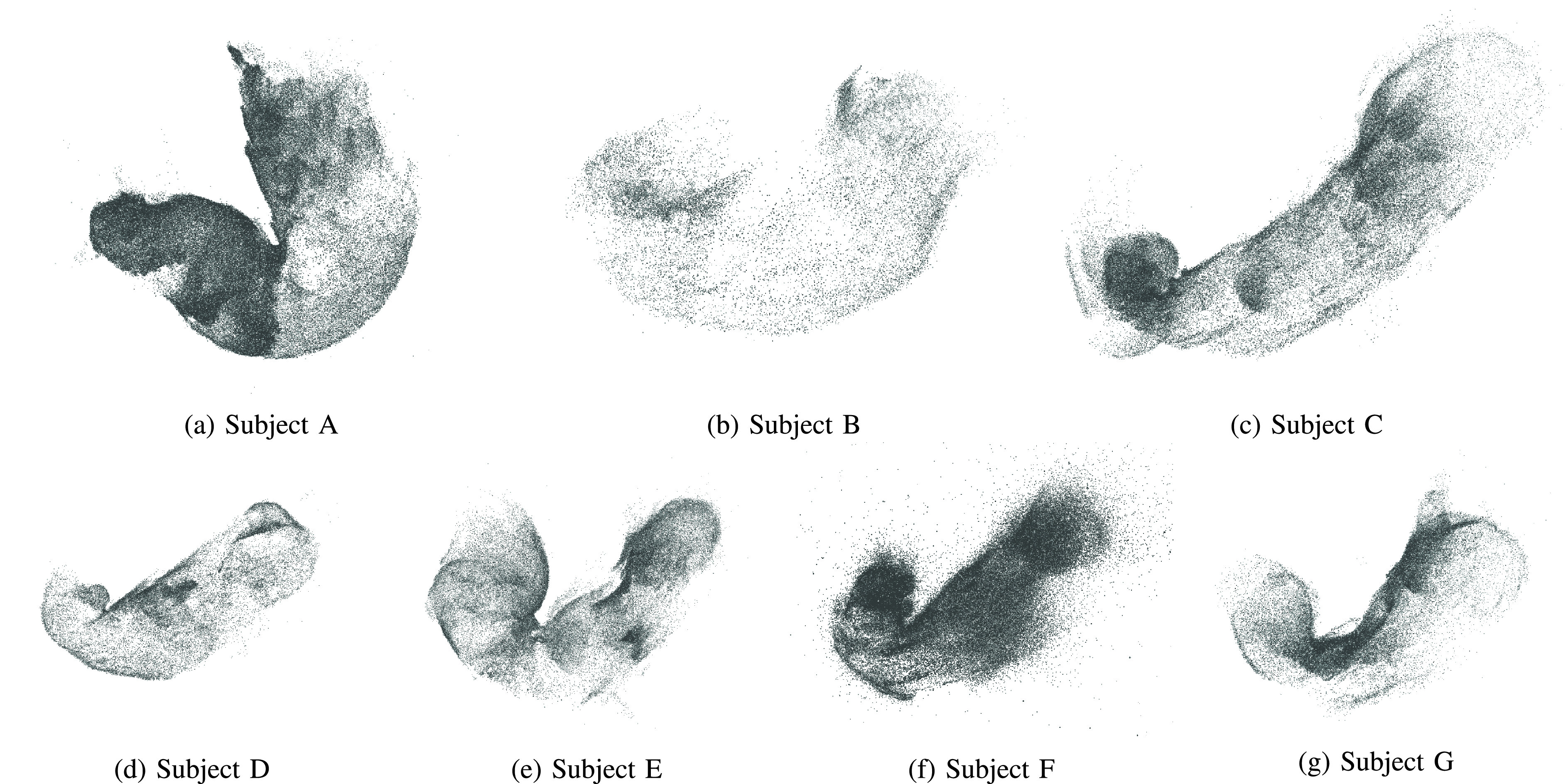
The point cloud reconstruction results with outlier removal obtained using the VIC red images from cGAN_g2r_. We can confirm that all the obtained point clouds resemble the shape of a stomach.

**FIGURE 10.**
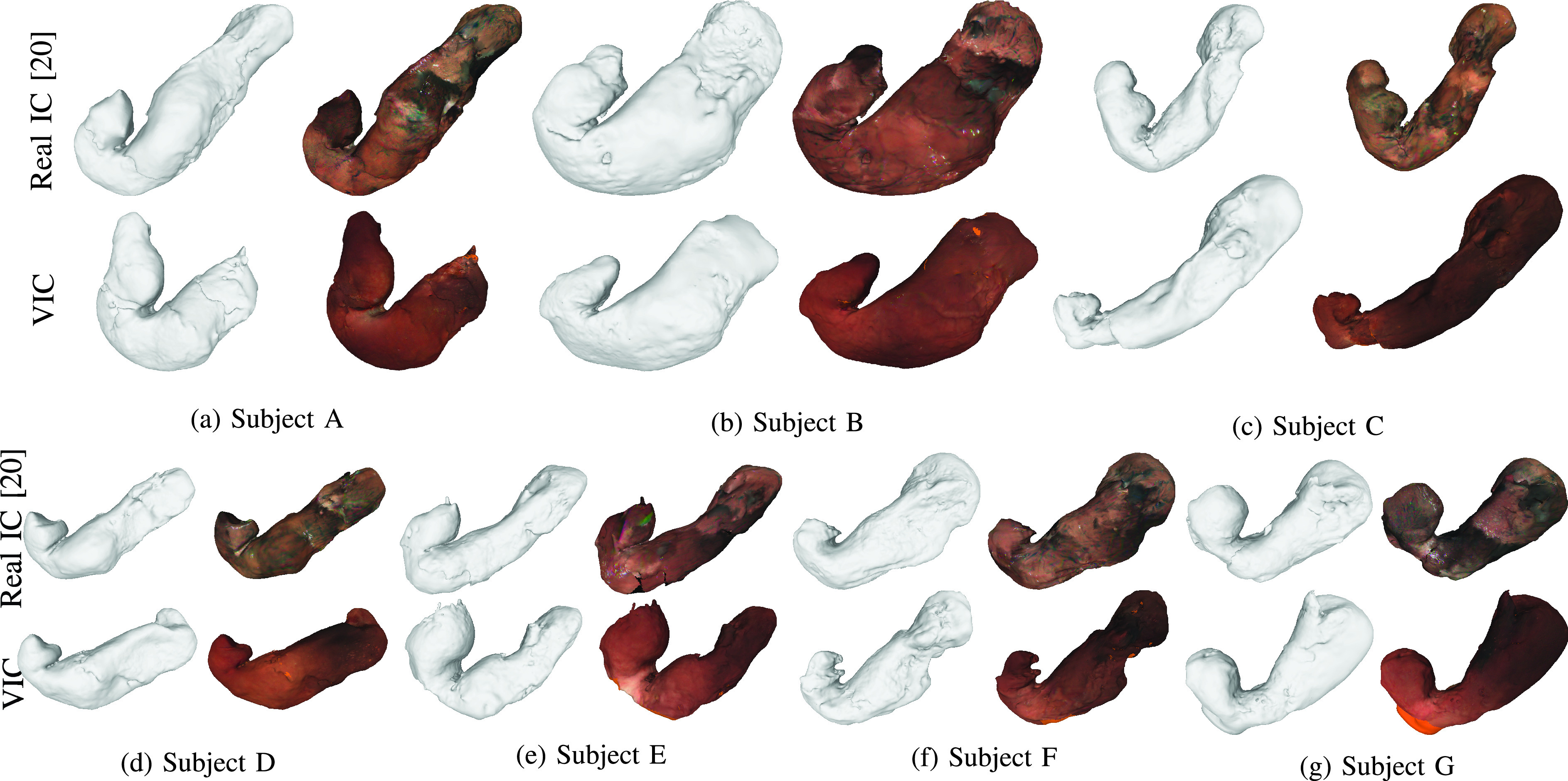
Visual comparison of the obtained mesh and texture models using VIC red images from cGAN_g2r_ (bottom row) and using real IC red images (top row). Since the input image sequences for each subject were captured at different time, there may be change in the stomach shape. In overall, the shapes and the characteristics are close to each other.

Figure 9 shows the point cloud results obtained with the whole stomach reconstruction pipeline using the VIC red images from cGAN_g2r_. We can see that the resulting point clouds are well reconstructed and resemble the shape of a stomach. Unfortunately, it is difficult to obtain ground-truth stomach 3D models for validation. While it is technically possible to obtain the 3D CT scan model of the stomach, the CT scan and endoscopy cannot be performed at the same time. Hence, the stomach could have significant difference in shapes. Because of that, we validate our reconstruction results by comparing them with the reconstruction results obtained using real IC red images as in [20] since the real IC and no-IC sequences were captured at the same endoscopy operation. Figure 10 shows the comparison of the reconstructed 3D mesh models obtained using VIC red images from cGAN_g2r_ and real IC red images. Since the input sequences used for each model reconstruction were captured at different time, some stomach movements were inevitable and the coverage area also may be different for each sequence. Even though this may cause some differences of the obtained 3D stomach models, we can see that the obtained models using VIC red images from cGAN_g2r_ capture the same overall structures as the models obtained using real IC red images.

**FIGURE 11.**
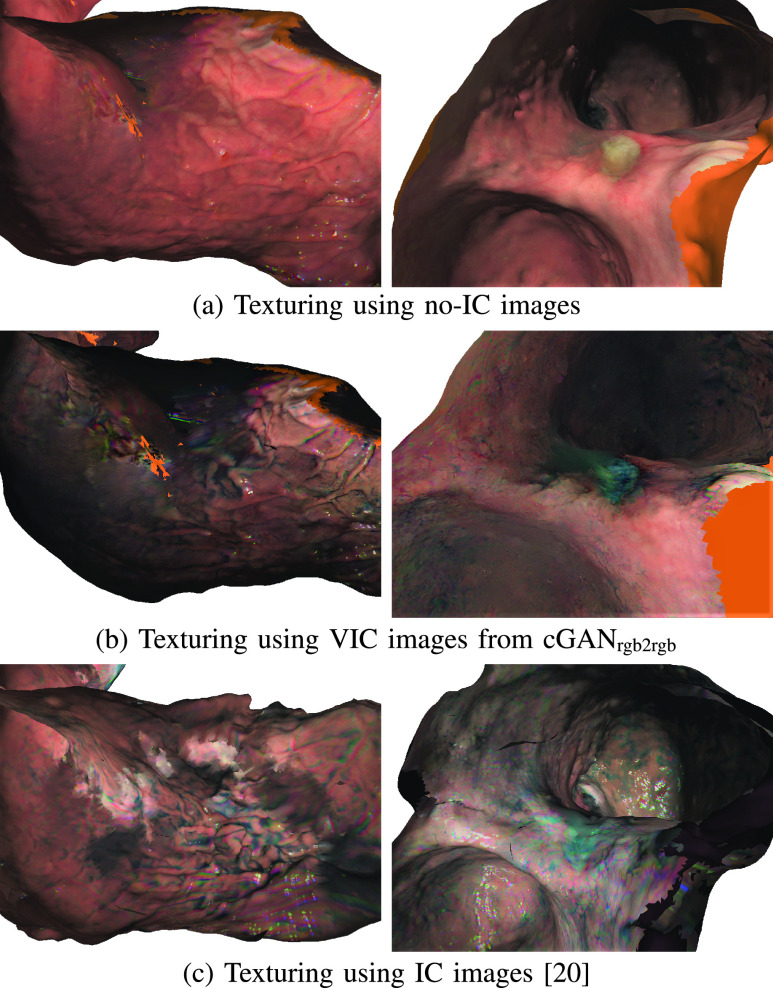
The images of (a) show the texturing results using no-IC images, the images of (b) show the texturing results using VIC images from cGAN_rgb2rgb_, and the images of (c) show the texturing results using real IC-sprayed images for comparison. Our proposed method allows us to use either no-IC or VIC texturing depending the purpose of the inspection.

One of the advantages of reconstructing the whole stomach using VIC images is that the texturing can be performed using either the original no-IC or the VIC RGB images. Figure 11 illustrates the difference between no-IC, VIC, and IC image texturing results on two subjects. Since there is no IC dye when capturing the real no-IC images, the textured mesh displays the gastric mucosa with natural color tone. Since the basic and general inspection to screen the whole stomach for lesion detection are performed using white light endoscopy, no-IC texture, in which there is no accumulated IC dye that hinders the visibility, is preferred for general screening. If there is any detected lesion, VIC texture can be used to enhance the lesion border and feature to investigate the lesion in more detail.

### E. Frame Localization and Local Refinement

**FIGURE 12.**
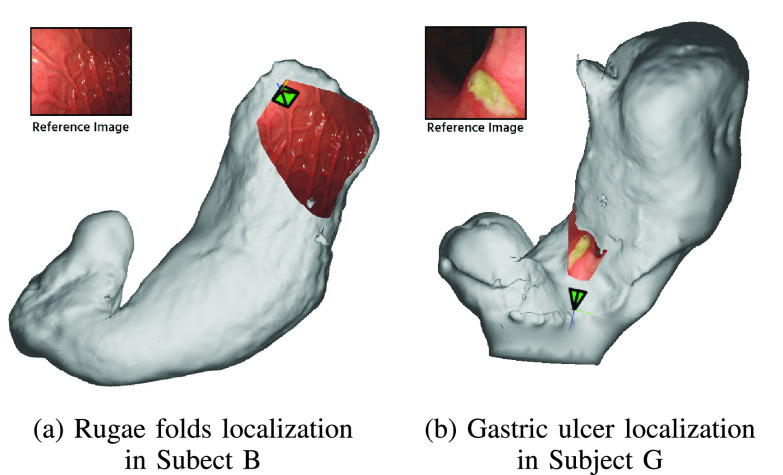
Two examples of the frame localization. An input reference image was selected from the list of reconstructed images. Then, the selected image’s camera pose (shown by the green pyramid) was obtained and the image texture was projected to the reconstructed mesh. We can see that, the relative position of the selected image to the whole stomach can effectively be identified and visualized.

Figure 12 shows two frame localization examples for Subject B and Subject G, where we used the real no-IC RGB image as an input to our frame localization. Figure 12(a) shows the frame localization of a rugae fold region. Figure 12(b) shows the frame localization of a gastric ulcer region. In Figure 12, we can see that the selected reference images are projected correctly to the reconstructed mesh and the relative position of the selected image to the whole stomach can be effectively identified and visualized.

**FIGURE 13.**
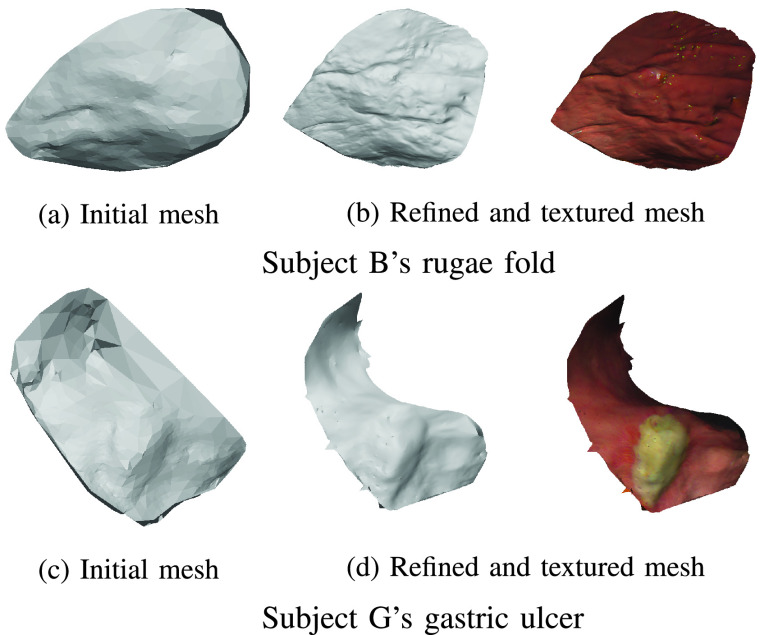
The result of our local refinement pipeline. Images (a)–(c) show the comparison between the initial and refined meshes for localized rugae fold using the input image in Figure 12(a). Images (d)–(f) show the comparison between the initial and refined meshes for localized gastric ulcer using the input image in Figure 12(b). We can see that while the initial mesh only produces a flat and low resolution mesh, our refined mesh has more refined details.

Figure 13 illustrates the results of our local mesh refinement. It shows the comparison between the low-resolution initial mesh generated by applying Poisson surface reconstruction and the refined mesh. Since our local refinement extracts the camera poses and the 3D points information from the global reconstruction, the obtained local structure is consistent with the global structure. We can see that the refined mesh by our proposed pipeline has better details compared to the initial mesh. It is clear that the rugae fold is visible in the refined mesh (Figure 13(b)) while it is not visible in the initial mesh (Figure 13(a)). The refined mesh has more detailed morphological information compared to the the initial mesh only showing the flat surface.

## IV. Conclusion

In this paper, we have presented a new approach to reconstruct a whole stomach 3D shape from a gastric endoscopy video without the need of IC dye spraying. We have applied CycleGAN as an image-to-image style translator to generate VIC red images from no-IC images for the stomach 3D reconstruction and shown that the generated VIC images significantly increase the number of extracted SIFT feature points. Furthermore, we have found that input color channel selection for the style translation affects the feature matching performance of the VIC images. Based on the investigation, we have found that the translation from no-IC green-channel images to IC-sprayed red-channel images gives significant improvements to the SfM reconstruction quality and completeness. We have experimentally demonstrated that our new approach can reconstruct the whole stomach shapes of all seven subjects and showed that the estimated camera poses can be used for the frame localization purpose. To validate our reconstruction results obtained using VIC red-channel images, we compared them with the reconstruction results obtained using real IC red-channel images and have shown that reconstructed stomach structures are similar to each other. In addition, we also presented a new local mesh refinement pipeline that is able to obtain a high-resolution textured mesh of an interesting local region for better inspection. For future works, we will be focusing on real-time whole stomach reconstruction by combining our VIC image generation and real-time depth and pose prediction as performed in deep-learning-based SLAM methods. We are also considering to combine our image-to-image translation with feature extraction and matching network learning for better image generation for the 3D reconstruction purpose. Finally, the videos of the reconstruction results can be accessed from the following link (http://www.ok.sc.e.titech.ac.jp/res/Stomach3D/).
